# An Asymptomatic Intrathoracic Kidney Secondary to a Bochdalek Hernia in a Geriatric Patient

**DOI:** 10.7759/cureus.86807

**Published:** 2025-06-26

**Authors:** Marielle Roberts-McDonald, Esteban Tapias, Muhammad Ali Khalid, Peter DeVito

**Affiliations:** 1 General Surgery, Ross University School of Medicine, Bridgetown, BRB; 2 General Surgery, Western Reserve Health Education/NEOMED Program, Warren, USA; 3 Internal Medicine, Western Reserve Health Education/NEOMED Program, Warren, USA; 4 Surgery, Western Reserve Health Education/NEOMED Program, Warren, USA

**Keywords:** adult diaphragmatic hernia, bochdalek hernia, diaphragmatic hernia, ectopic kidney, intrathoracic kidney

## Abstract

A Bochdalek hernia is a congenital defect that permits abdominal organs to herniate into the thoracic cavity. While commonly diagnosed in neonates due to symptomatology, it is often found incidentally in adults. The case presents an 84-year-old male from a skilled nursing facility complaining of left-sided chest pain radiating to the left shoulder, accompanied by shortness of breath and nausea. Initially worked up for an acute coronary cause or pulmonary embolism, imaging revealed an incidental right-sided Bochdalek hernia with intrathoracic displacement of the right kidney. Given the absence of gastrointestinal or pulmonary symptoms, the patient was conservatively managed. While surgical repair is preferred for symptomatic Bochdalek hernias in adults, conservative management is a viable option for stable patients. This case highlights the importance of incidental imaging findings in guiding clinical decision-making and patient-focused care.

## Introduction

Bochdalek hernia is a congenital defect characterized by an opening in the posterior lateral diaphragm that allows abdominal contents to protrude into the thoracic cavity, most commonly seen in children [1,2]. Children within the first several weeks of life present with respiratory distress due to mechanical compression of the developing lung parenchyma [1]. In adults, Bochdalek hernias are rare, especially in symptomatic adults, and are found incidentally on imaging in asymptomatic adults [1]. Specifically, Bochdalek hernias are an extremely uncommon occurrence and even more rare to be located on the right side due to the pleuroperitoneal canal closing first in the embryo on the right side [1]. The reported incidence in asymptomatic adults ranges from 0.17% to 6%, with questionable pathophysiology of development during adulthood; however, there are reports of >35% incidence when greater than 70 years old [1,3].

There are approximately 100-150 reports of Bochdalek hernias found in adults due to the incidental nature [2]. When Bochdalek hernias are symptomatic, there are concerns for complications including incarcerated bowel, intra-abdominal organ dysfunction, and severe pulmonary disease [2]. Surgical management is recommended, either laparoscopy, thoracoscopy, primary closure, or mesh repair [4]. It is unclear why, however, postoperatively, patients are at risk for cardiopulmonary complications, including cardiac arrest or chronic obstructive pulmonary disease [4]. Patients may experience empyema due to lung re-expansion pulmonary edema, which was observed in a case with ischemic or necrotic changes in the herniated abdominal viscera [4].

The case presented below is an 84-year-old male from a skilled nursing facility who presented with left-sided chest pain radiating to his shoulder and who has incidentally found that his right kidney was located within the thorax due to a Bochdalek hernia.

## Case presentation

The patient is an 84-year-old male from a skilled nursing facility with pertinent past medical history. This includes coronary artery disease (myocardial infarction with two stents, last ejection fraction 59%) on clopidogrel 75 mg, aspirin 81 mg, and metoprolol 25 mg; obstructive sleep apnea; dementia; type 2 diabetes on insulin aspart 100 unit/mL subcutaneous; and gastroesophageal reflux disease on omeprazole 20 mg. He presented to the emergency department (ED) complaining of left-sided chest pain radiating to the left shoulder. The patient reported that the pain started yesterday afternoon and has been constant. He was awoken by the chest pain, prompting ED evaluation. The patient also admitted to shortness of breath, nausea, and generalized body aches. He denied abdominal pain, palpitations, and bowel changes. His last cardiac catheterization was about nine months ago.

**Figure 1:**
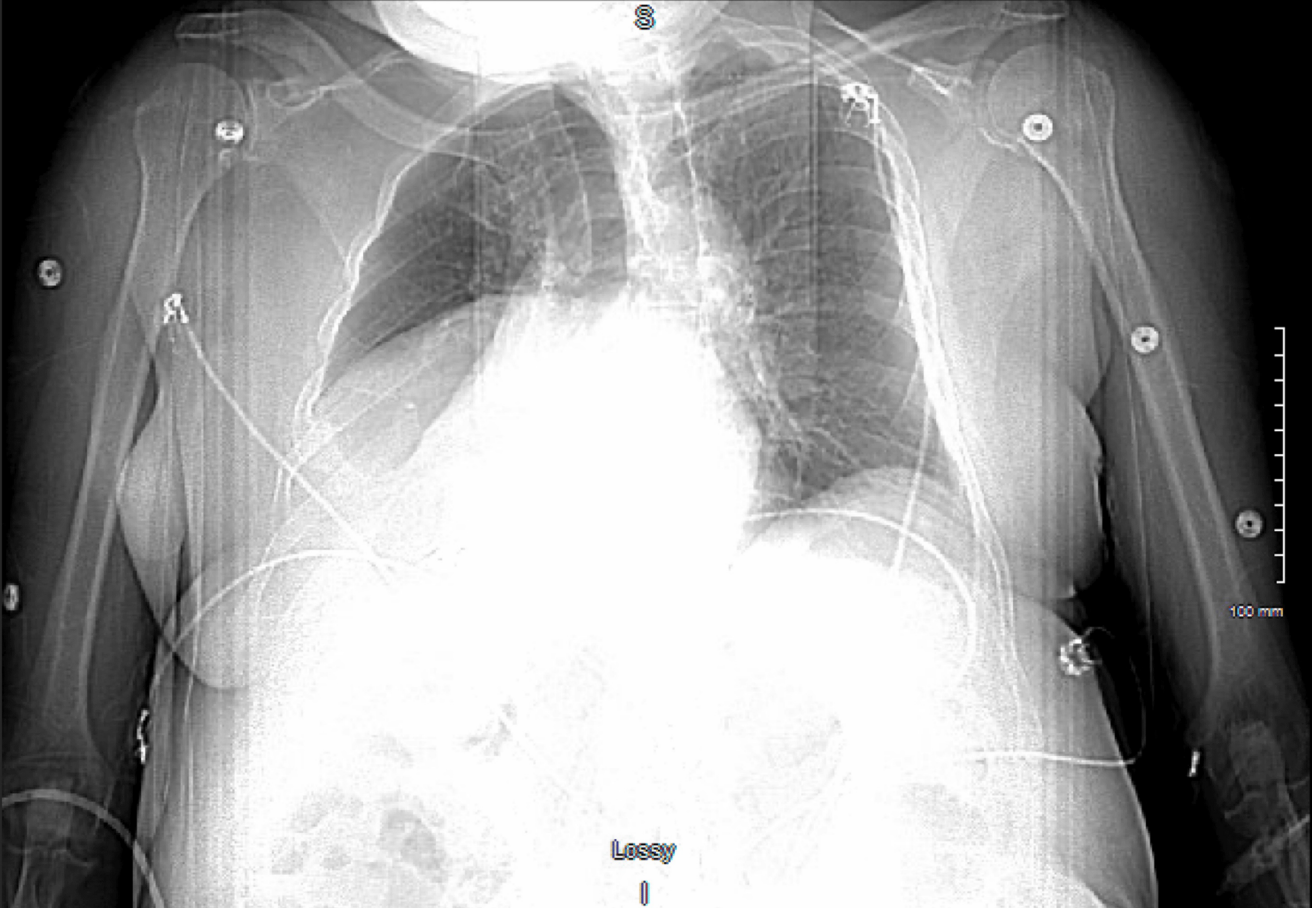
Chest X-ray demonstrating no evidence of acute cardiopulmonary disease

The patient was stable, with a heart rate of 56 beats per minute, blood pressure of 204/84, respiratory rate of 18 breaths per minute, 98% oxygen saturation on room air, and temperature of 97.6°F. Labs demonstrated elevated D-dimer of 1.76 and troponin of 35, 30, and 22 (seen in Table 1). Electrocardiogram demonstrated sinus bradycardia at 52, first-degree block, right bundle branch block, and inferior T-wave inversion; compared to EKG nine months ago, inferior T-wave inversions appeared new; otherwise, no acute changes. Chest X-ray (seen in Figure 1) demonstrated no evidence of acute cardiopulmonary disease and no significant change in right hemidiaphragm elevation. The patient was given aspirin 324 mg PO once in the ED. He was admitted to medicine for chest pain (moderate coronary artery risk).

**Table 1:** Laboratory parameters of the initial emergency department visit BUN: blood urea nitrogen

Laboratory Parameters	Patient Values	Reference Range
White Blood Cells	5.0 x 10E3/uL	3.5-10.5 x 10E3/uL
Hemoglobin	13.2 gm/dL	13.5-17.5 gm/dL
Platelet Count	194 x 10E3/uL	150-450 x 10E3u/L
Sodium	142 mmol/L	137-146 mmol/L
Potassium	3.5 mmol/L	3.5-5.3 mmol/L
Chloride	103 mmol/L	98-107 mmol/L
Glucose	87 mg/dL	<100 mg/dL
Creatine	1.0 mg/dL	0.6-1.4 mg/dL
BUN	14 mg/dL	5-25 mg/dL
Troponin	35 ng/L	<14 ng/L
D-Dimer	1.76 ug/mlFEU	<0.50 ug/mlFEU

**Figure 2:**
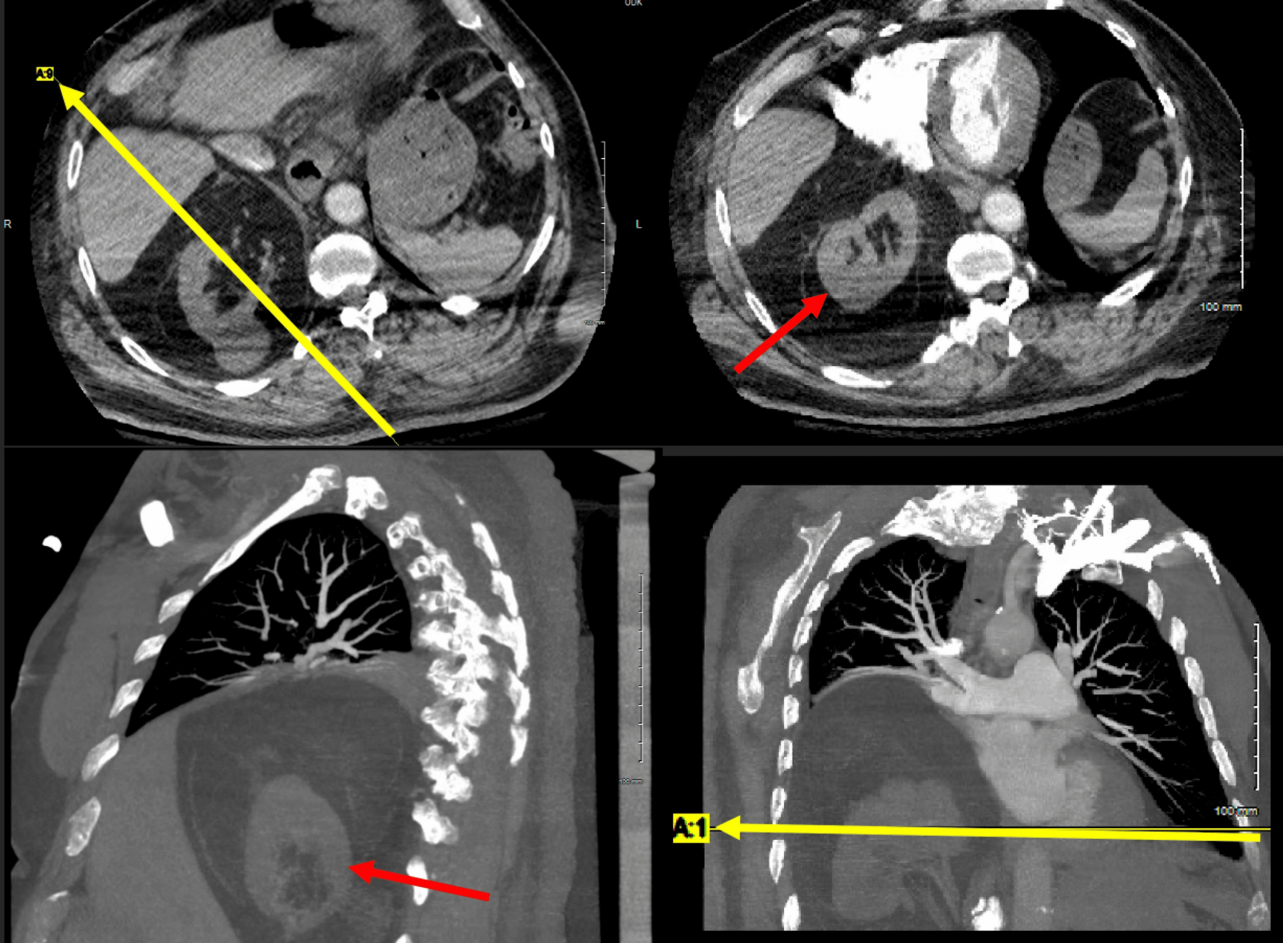
Chest CTA demonstrating marked elevation of the right hemidiaphragm with retroperitoneal fat and the right kidney located within the thorax Top left: Axial CT scan, with a yellow arrow line indicating the plane of the corresponding sagittal view (bottom left). Bottom left: Sagittal CT view, with a red arrow marking the ectopic kidney. Top right: Axial CT scan, with a red arrow marking the ectopic kidney. Bottom right: Coronal CT view, with a yellow arrow line indicating the image plane from the axial scan above (top right).

During admission, cardiology was also consulted due to moderate coronary artery risk and recommended a pharmacological nuclear stress test. The stress test demonstrated left ventricular ejection fraction calculated at 64%, and no acute ischemia was noted. Chest CTA (seen in Figure 2) was also ordered to rule out pulmonary embolism due to elevated D-dimer. Chest CTA demonstrated the following: (1) no evidence of pulmonary embolism to the subsegmental level; (2) marked elevation of the right hemidiaphragm with retroperitoneal fat and the right kidney located within the thorax; (3) dependent atelectasis within the right middle and lower lobes; and (4) amall hiatal hernia. The patient was then discharged the next day back to the nursing facility and recommended to follow up with cardiology and the primary care physician.

## Discussion

Bochdalek hernia is a diaphragmatic defect, rarely diagnosed in asymptomatic adults. The case presented above of an 84-year-old male was recommended for both a cardiology and pulmonology work-up when a chest CTA was performed, demonstrating a diaphragmatic hernia in the R thoracic cavity with their kidney displaced superiorly.

Symptomatic Bochdalek hernia can be a life-threatening concern, specifically when the patient presents with gastrointestinal symptoms pointing toward obstructive signs that may involve a herniated organ [2]. When it is a left-sided hernia, organs of concern include the colon, stomach, spleen, small bowel, pancreas, and even the adrenal gland [2]. When compared to a right-sided hernia, there is less of a concern due to possible involvement of the liver or kidney herniating [2]. Pulmonary symptoms are less common when compared to gastrointestinal ones, where the patient may present with shortness of breath or recurrent chest infections [2]. Overall, the incidence of a right-sided Bochdalek hernia is rare, and right-sided intrathoracic kidney herniation into a Bochdalek hernia is even rarer [2].

One case reported a 26-year-old male presenting with abdominal pain and constipation diagnosed with an ectopic intrathoracic kidney secondary to a diaphragmatic hernia, where conservative management was decided [5]. Based on the Pfister-Goedeke and Brunier’s classification of renal ectopia, both the patient presented above and the 26-year-old are a part of the less than 0.25% statistic of patients with category type 3, ectopic kidney due to diaphragmatic hernia [5]. Due to the asymptomatic nature of the 26-year-old. The patient decided to be managed conservatively, similar to the case presented above [5]. The conservative management included monitoring for progression of symptoms and monitoring for complications such as pulmonary hypertension or renal failure [5]. Although most patients with this abnormality are treated surgically, the patient’s request should be considered during medical decision-making when offering the best treatment [5]. In this case report, three other patients were also conservatively managed and reported no changes or symptoms after discharge [5].

## Conclusions

This case highlights the rarity of a Bochdalek hernia in adults without clinical compromise. The case underscores the importance of considering diaphragmatic hernias when a patient presents with atypical chest pain. Incidental findings are important to be considered in clinical management due to the symptomatology seen in adults. The elderly patient above presented with chest pain, and imaging demonstrated an unexpected right-sided Bochdalek hernia with intrathoracic kidney displacement, with no clinical compromise noted. This case emphasizes the role of imaging in diagnosing incidental diaphragmatic hernias and supports individualized treatment approaches based on symptom severity and risk of complications.
